# Chronic *Opisthorchis viverrini* Infection Changes the Liver Microbiome and Promotes *Helicobacter* Growth

**DOI:** 10.1371/journal.pone.0165798

**Published:** 2016-11-02

**Authors:** Upsornsawan Itthitaetrakool, Porntip Pinlaor, Somchai Pinlaor, Chariya Chomvarin, Rungtiwa Dangtakot, Apisit Chaidee, Chotechana Wilailuckana, Arunnee Sangka, Aroonlug Lulitanond, Puangrat Yongvanit

**Affiliations:** 1 Biomedical Science Program, Graduate School, Khon Kaen University, Khon Kaen, Thailand; 2 Centre for Research and Development of Medical Diagnostic Laboratories, Faculty of Associated Medical Sciences, Khon Kaen University, Khon Kaen, 40002, Thailand; 3 Department of Parasitology, Faculty of Medicine, Khon Kaen University, Khon Kaen, 40002, Thailand; 4 Science Program in Medical Technology, Faculty of Associated Medical Sciences, Khon Kaen University, Khon Kaen, 40002, Thailand; 5 Department of Microbiology, Faculty of Medicine, Khon Kaen University, Khon Kaen, 40002, Thailand; 6 Department of Biochemistry, Faculty of Medicine, Khon Kaen University, Khon Kaen, 40002, Thailand; 7 Liver Fluke and Cholangiocarcinoma Research Center, Khon Kaen University, Khon Kaen, 40002, Thailand; Instituto Butantan, BRAZIL

## Abstract

Adults of *Opisthorchis viverrini* reside in the biliary system, inducing inflammation of bile ducts and cholangitis, leading to hepatobiliary disease (HBD) including cholangiocarcinoma. *O*. *viverrini* infection also has major implications for the bacterial community in bile ducts and liver. To investigate this in chronic *O*. *viverrini* infection (≥ 8 months p.i.), bacterial genomic DNA from livers of hamsters and from worms was investigated using culture techniques, PCR for *Helicobacter* spp. and high-throughput next-generation sequencing targeting the V3-V4 hypervariable regions of prokaryotic 16S rRNA gene. Of a total of 855,046 DNA sequence reads, 417,953 were useable after filtering. Metagenomic analyses assigned these to 93 operational taxonomic units (OTUs) consisting of 80 OTUs of bacteria, including 6 phyla and 42 genera. In the chronic *O*. *viverrini*-infected group, bacterial community composition and diversity were significantly increased compared to controls. Sequences of *Fusobacterium* spp. were the most common (13.81%), followed by *Streptococcus luteciae* (10.76%), *Escherichia coli* (10.18%), and *Bifidobacterium* spp. (0.58%). In addition, *Helicobacter pylori* (0.17% of sequences) was also identified in the liver of chronic *O*. *viverrini* infections, but not in normal liver. The presence of *H*. *pylori* was confirmed by PCR and by use of an antibody against bacterial antigen, supporting the metagenomics data. The identities of bacteria cultured for enrichment suggested that chronic *O*. *viverrini* infection changes the liver microbiome and promotes *Helicobacter* spp. growth. There may be synergy between *O*. *viverrini* and the liver microbiome in enhancing immune response-mediated hepatobiliary diseases.

## Introduction

Opisthorchiasis, caused by *Opisthorchis viverrini* infection, remains a major health problem in the Greater Mekong Subregion, including Thailand, Laos, Vietnam and Cambodia. This carcinogenic food-borne trematode has a high prevalence in northeastern Thailand, where approximately 6 million people are currently infected [1, 2]. Humans acquire the infection by eating undercooked cyprinid fish, which are contaminated with infective stage metacercariae. Metacercariae excyst in the duodenum and juvenile worms migrate into the hepatobiliary system. Worms reach adulthood after four weeks and produce eggs that are voided in feces. If ingested by a *Bithynia* snail, the egg hatches and undergoes transformation and multiplication to release many cercariae that penetrate the skin of freshwater cyprinid fish.

Chronic infection with *O*. *viverrini* can cause hepatobiliary diseases (HBD) including cholangitis, periductal fibrosis, cholecystitis, obstructive jaundice and cholangiocarcinoma (CCA) [1, 3]. Approximately 10% of opisthorchiasis patients are likely to develop CCA [4]. Nowadays, although the incidence of opisthorchiasis is declining due to a multi-faceted control program [2], the incidence of CCA in endemic areas of liver fluke, such as in Khon Kaen Province, remains high [5] and the incidence of CCA elsewhere is increasing [6]. This suggests the etiology of HBD and CCA are associated with multifactorial risk factors [7].

Although a precise mechanism leading to opisthorchiasis-associated CCA is not fully known, pathogenesis is likely to be influenced by a diet rich in nitrosamine, chronic inflammation of bile ducts and molecules secreted by parasites [1, 3]. Periductal fibrosis in chronic liver fluke infection, or partial obstruction of ducts by flukes, may affect bile flow and bile composition, leading to enhanced bacterial growth in the hepatobiliary tract [8].

Besides liver flukes, bacterial infection can participate in cholangitis [8]. To date, however, there is little information concerning the influence of *O*. *viverrini* infection on the liver and HB tract microbiome of the host. The colorectal and biliary microbiomes have recently been investigated in experimental opisthorchiasis [9]. Several *Helicobacter* species have been identified from the gallbladders of Syrian hamsters in association with cholangiofibrosis and centrilobular pancreatitis [10] as well as in precancerous lesions before CCA development [11]. Moreover, genomic DNA of *Helicobacter pylori* has been identified in CCA tissues from Thai patients [12]. In humans and rodents, HBD is not only caused by *H*. *pylori*, but also by other species of *Helicobacter* [13]. We therefore hypothesize that bacterial infection may participate in induction of HBD during liver fluke infection.

The present study aimed to identify the microbiome in livers of hamsters with chronic opisthorchiasis. Several techniques were used including culture for aerobic bacteria, PCR for *Helicobacter* spp. and metagenomic analysis of portions of the 16S rRNA gene generated using high-throughput next-generation sequencing [14–16]. We found considerable bacterial diversity and identified *H*. *pylori* in the livers of hamsters with chronic opisthorchiasis. We provide novel information of co-infection between bacteria and *O*. *viverrini* in the hepatobiliary system.

## Materials and Methods

### Ethics statement

All procedures involving animals were carried out based on recommendations in the Guide for the Care and Use of Laboratory Animals of the National Research Council of Thailand. All surgery and necropsy was performed under ether anesthesia, and every effort was made to minimize pain and suffering to the animals. Food, water and general animal health were checked daily. Animal bedding was changed twice a week. The study protocol, including the use of ether, was approved three years ago by the Animal Ethics Committee of Khon Kaen University (Permit Number: 0514.1.12.2/59, in 2013).

### Experimental animals

Twenty-six male Syrian golden hamsters (*Mesocricetus auratus*), aged between 4 and 6 weeks, were used. The animals were divided into 2 groups: 1) normal hamsters (Normal, n = 12, 5 animals necropsied at 8 months and 7 animals at 15 months), and 2) chronic *O*. *viverrini*-infected group (OV, n = 14, 5 animals necropsied at 8 months post-infection (p.i.), 1 animal at 12 months p.i. and 8 animals at 15 months p.i.). Hamsters in the OV group were each infected with 50 *O*. *viverrini* metacercaria by oral inoculation.

*Opisthorchis viverrini* metacercaria were isolated from naturally infected cyprinoid fishes, collected from endemic areas, by artificial pepsin digestion in 0.25% Pepsin A (BDH, USA) solution as described elsewhere [17].

### Specimen collection

**Fig 1:**
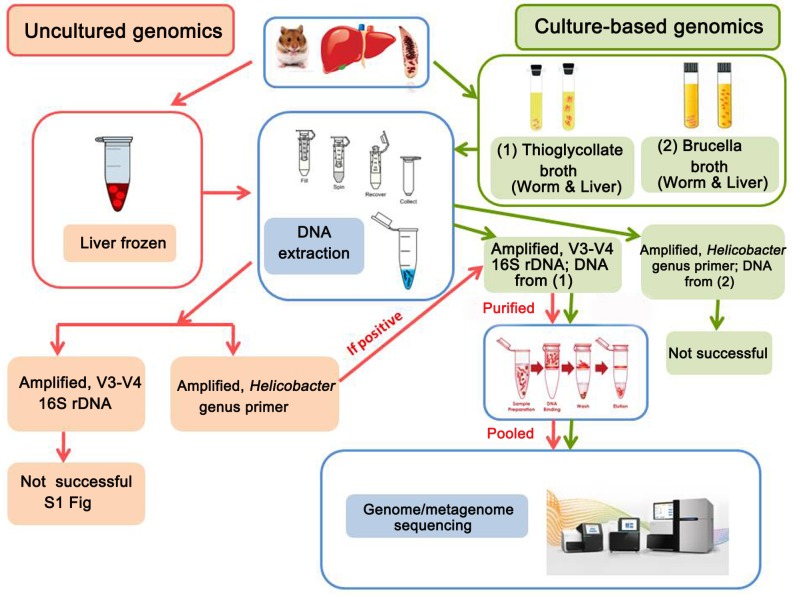
Flow diagram showing the approaches used to obtain genome sequences from cultured and uncultured microbes. Pink lines show the sequence of manipulations for frozen samples, and green lines for culture-based samples.

A flow chart of sample preparation methods leading to metagenomic analysis is shown in Fig 1. For necropsy, hamsters were anesthetized with ether. Liver tissue at the hilar region and containing a large bile duct was immediately collected and snap-frozen in liquid nitrogen and then stored at -20°C until analysis. Additional liver samples were collected and cultured in both thioglycollate and Brucella broths. A final sample was fixed in 10% buffered formalin for histopathological study and immunohistochemistry. In addition, intact adult worms were randomly collected from the gallbladder and biliary systems for cultivation.

### Bacterial cultivation

To enhance all bacterial growth, infected and normal liver tissues obtained from all experimental samples were cultured in thioglycollate broth supplemented with 20% fetal bovine serum using sterile technique for at least three days. In order to identify aerobic bacteria, broth was plated on blood agar and MacConkey agar. After incubation for two days, a single colony from either of those agar plates was inoculated on various biochemical tests as described elsewhere [18] for Gram-negative bacteria (oxidase test, triple sugar iron, lysine, Simmons citrate, urease test, motile/indole) and Gram-positive bacteria (starch, *Streptococcus faecalis* broth, bile esculin, sensitivity to bacitracin and optochin). Identification of aerobic bacteria was based on morphology of the colony on an agar plate and biochemical test outcomes.

To enhance growth of *Helicobacter* species, liver samples (infected and normal) were cultured in Brucella broth supplemented with 10% fetal bovine serum + 3.5 mM H_2_O_2_ for at least 7 days as described elsewhere [19].

In addition, worm (5 worms/ broth) obtained from 5 infected hamsters at 15 months post-infection were cultured in both thioglycollate broth and Brucella broth for at least three days. After centrifugation, the sediments from broths were used for DNA extraction.

### Preparation of microbial genomic DNA (gDNA)

gDNA was extracted directly from frozen liver and after culture of all experimental samples in thioglycollate broth and in Brucella broth. gDNA was also extracted from pools (5 worms) of cultured intact adult worms obtained from 5 infected hamsters at 15 months post-infection. DNA extraction was performed using a QIAamp Tissue Kit (Qiagen, Germany) according to the manufacturer’s protocol. Concentration, purity and integrity of the gDNA were determined by spectrophotometry (Nanodrop 2000; NanoDrop Technologies, Wilmington, DE, USA). The gDNAs were stored at −80°C for further study.

### Amplification of 16S rRNA genes, *Helicobacter* genus-specific gene and *H*. *pylori ureA* gene

The prokaryotic 16S rRNA (V3-V4 region) [20] was amplified from directly frozen samples and from material after culture in thioglycollate broth using V3-V4 primers as presented in Table 1. After amplification, the PCR products were purified individually using a clean-up kit (Gene JET PCR Purification kit, Thermo Scientific). Quality and concentration of amplified DNA were checked using gel electrophoresis. PCR products from individual samples were pooled by group and stored at −80°C until analysis.

**Table 1:** List of primers and conditions for PCR reactions used to amplify a particular target of bacteria regions.

Organism	Genes	Primer sequence (5’→3’)	Product size (bp)	PCR conditions
Prokaryotebacteria	16S rDNA (V3-V4) Pro341-FPro802-R	5’CCTACGGGNGGCWGCAG3’5’TACNVGGGTATCTAATCC3’	459	94°C 5 mins, 94°C 40 sec, 52.8°C 30 sec, 72°C2 mins, 35 cycles, 72°C 10 mins
*Helicobacter* genus-level	16S rDNAC97-FC98-R	5’GCTATG ACG GGT ATC C3’5’GATTTT ACC CCT ACA CCA3’	400	94°C 5 mins, 94°C 1 mins,57°C 1.5 mins,72°C 1 min, 35 cycles, 72°C 7 mins
*Helicobacter pylori*	*ureA*-F*ureA*-R	5’AGTTCCTGGTGAGTTCTTAA3’5’AACCACGCTCTTTAGCTCTGTC3’	350	94°C 2 mins, 94°C 30 sec, 55.7°C 30 sec, 72°C 1 min, 40 cycles,72°C 5 mins

Attempts to amplify the V3-V4 region directly from DNA from frozen liver were unsuccessful. However, three of the frozen samples yielded a band when amplified using *Helicobacter* genus-specific primers (C97 and C98). These PCR products, when re-amplified using the V3-V4 region primers yielded new bands (Fig 1, S2 Fig). *Helicobacter pylori* DNA (LMG 8775 DMST 20165 type strain) was obtained from the National Institute of Health, Department of Medical Sciences, Ministry of Public Health, Thailand and was used as a positive control. PCR amplification was performed with a thermal cycler and an Expand High-Fidelity PCR system (Bio Rad C100^™^thermal cycler). Each reaction (20 μl) contained 1× Expand High-Fidelity buffer, 1U Platinum *Taq* DNA polymerase, a 5 μM concentration of primers, a 10 mM concentration of each deoxyribonucleotide triphosphate, and 50 mM MgCl_2_. Amplification conditions are shown in Table 1.

Liver samples yielding a positive PCR result for the genus *Helicobacter* were further investigated for the presence of *H*. *pylori* using species-specific *ureA* gene primers following the protocol in Table 1.

### Next-generation sequencing, sequence processing and OTU-based analysis

Four pools of purified PCR products containing amplicons of the V3-V4 region were used as follows: 1) pooled normal hamster at 15 months, 2) pooled positive PCR products from OV-infected hamsters at 15 months p.i., 3) pooled *Helicobacter* genus-specific amplicons from frozen livers of OV-infected hamsters at 8 & 12 months p.i., and 4) pooled worms at 15 months p.i. These were submitted to next-generation sequencing followed by metagenomic analysis as described elsewhere [14–16]. The PCR products were sequenced using next-generation sequencing.

Paired-end reads were generated with the Illumina MiSeq platform (MiSeq Reagent Kit v3 (600 cycles). MiSeq Sequencing Reporter (MSR) was used for de-multiplexing of data and removal of reads that failed Illumina's purity/chastity filter (PF = 0) [21]. In order to obtain more accurate and reliable results in subsequent bioinformatics analysis, the raw data were pre-processed using an in-house (Beijing Genomics Institute; BGI, Co., Ltd., China) procedure as follows: 1) Truncation of sequence reads not having an average quality of 20 over a 25 bp sliding window based on the phred algorithm, and removal of trimmed reads and their pairs having less than 75% of their original length; 2) Removal of reads contaminated by adapter (default parameter: 15 bases overlapped by reads and adapter with maximal 3 bases mismatch allowed); 3) Removal of reads (including their pairs) with ambiguous bases (N); 4) Removal of reads with low complexity (default: reads with 10 consecutive identical bases). For the pooled libraries with barcode samples mixed, the clean reads were assigned to corresponding samples by allowing no base mismatch to barcode sequences using in-house scripts.

After the raw data were filtered as described above, paired-end reads with overlap were merged to create tags. All unique tags were clustered into Operational Taxonomic Units (OTUs) using scripts in USEARCH (v7.0.1090). OTUs were further filtered by 1) removal of unassigned OTUs and 2) removal of OTUs not assigned to the species. The filtered OTUs were used for downstream processing. Then OTU-representative sequences were classified to taxonomic levels (phylum, genus, species where possible) using Ribosomal Database Project (RDP) Classifier v.2.2 trained on the Greengenes Database (0.8 confidence values as cutoff). Finally, OTUs were analyzed in terms of presence/absence, abundance, or phylogenetic diversity [22].

#### Analysis of community patterns, taxonomic identification and diversity analysis

The sequences were assembled into OTUs with an identified threshold of ≥97% sequence similarity [23, 24] using UPARSE [25]. Chimeras were filtered out using UCHIME (v4.2.40) [26]. A representative sequence for each OTU was obtained using USEARCH GLOBAL, and then the number of sequences in each OTU calculated as an indication of OTU abundance. The indices were calculated by Mothur (v1.31.2) [23], and the corresponding rarefaction curve drawn using software R (v3.0.3) [27]. The formula used to calculate each index can be found at http://www.mothur.org/wiki/Calculators. The rarefaction curve was constructed as follows: 1) OTU numbers were calculated based on extracted tags (in multiples of 500); 2) the rarefaction curve was drawn using the indices calculated with extracted tags.

#### Heat map analysis

A heat map is a graphical representation of data where the individual values contained in a matrix are represented as different colors. Heat maps at the genus and species levels were created with the g-plots program of software R (v3.0.3) [27] and the distance algorithm is 'euclidean'. The clustering method is 'complete'. Clusterings of genera and species based on the abundance of each were shown by heat map at different taxonomic ranks. Vertical clustering indicates the similarity of all genera/species among different samples, and the horizontal clustering indicates the similarity of certain genera/species among different samples. The closer the distance is and the shorter the branch length is, the more similar the genus/species composition is between the samples. Any genus or species of which abundance is less than 0.5% in all samples was classified into 'others'.

The relative abundance values were all log transformed. If the relative abundance of certain species was 0, then half of the minimum abundance value was substituted for it.

#### Phylogenetic tree analysis

A phylogenetic tree is a branching diagram showing the inferred evolutionary relationships among various biological species or other entities (their phylogeny) based upon similarities and differences in their physical or genetic characteristics. The evolutionary distance between species is closer if the branch length is shorter. Representative sequences were aligned against the Silva core set (Silva_108_core_aligned_seqs) using PyNAST by 'align_seqs.py' [28]. A phylogenetic tree of representative OTUs was constructed using scripts built-in to QIIME (v1.80) [29] including the fast tree method for tree construction. Taxonomic ranks were assigned to OTU representative sequences using the Ribosomal Database Project (RDP) Bayesian Classifier v.2.2 [30]. Finally, alpha diversity and beta diversity were analyzed based on OTU and taxonomic ranks. The tag with the highest abundance within each genus was chosen as the corresponding genus representative sequence, and a genus-level phylogenetic tree was obtained in the same way as the OTU phylogenetic tree. The phylogenetic tree was imaged by software R (v3.0.3).

### Immunohistochemistry study

In order to detect the presence of *H*. *pylori* in liver tissue, liver paraffin sections (5-μm thickness) were deparaffinized in xylene and rehydrated in descending concentrations of ethanol. To enhance the immunostaining, the sections were placed in citrate buffer (pH 6.0) and autoclaved at 110°C for 10 minutes for retrieval of antigen. Next, the sections were transferred to 3% H_2_O_2_ in PBS buffer to inhibit endogenous peroxidase activity. Then, the sections were incubated with 5% fetal bovine serum in PBS for 30 minutes and incubated with rabbit polyclonal *H*. *pylori* antibody (Abcam, ab 7788; 1:10 dilution) at 4°C overnight. Finally, slides were incubated with the secondary antibody (peroxidase-conjugated goat anti-rabbit IgG) at room temperature for 1 hour. The stained sections were examined using a microscope.

### Statistical analysis

All statistical analyses were performed using the SPSS version 15 for Windows (SPSS Inc., Chicago, IL, USA). A *P*-value less than 0.05 was considered a significant difference.

## Results

### Identification of bacteria by culture

Aerobic bacteria, identified based on morphology and biochemical tests, were found in about 60% of livers (after culture in thioglycollate broth) from the OV-infected group. Bacterial taxa included *Escherichia coli* (40% of livers), *Streptococcus* group D *non Enterococci* (20%), *Enterobacter* spp. (20%), *Streptococcus pyogenes* (group A) (20%) and *Klebsiella pneumoniae* (20%). In contrast, no aerobic bacterial growth was seen in cultures of normal liver tissues. After culture in Brucella broth for at least 7 days, no bacterial growth was observed from infected liver tissues at 8 and 15 months p.i. or from normal liver tissues under the microaerophilic conditions used for culture of *Helicobacter* spp. One individual hamster was sampled at 12 months because it was sick. In addition, sediments from cultures of worms in both broths were used for DNA extraction, but biochemical tests to identify aerobic bacteria were not done.

### Detection of bacterial gDNA bacteria by PCR

From frozen liver samples no PCR amplicons were obtained for the prokaryotic 16S rDNA (V3-V4 region) from either normal or OV-infected groups at any time point p.i. (S1 Fig). However, three frozen liver samples of the OV-infected group (3/14, 22%) were positive at the genus level for *Helicobacter* (primers C97/C98) but normal livers and adult worms all returned negative results (S2 Fig). Attempted re-amplification of these three PCR products using the primers for the V3-V4 region appeared to be unsuccessful (the 16S rDNA primers were not nested within the region spanned by the *Helicobacter*-specific primers). Curiously, following NGS analysis of these three re-amplified samples, some sequences were returned that were from the V3-V4 region: some amplification of this region must have occurred.

After culture in thioglycollate broth, all liver samples in the chronic OV-infected group (14/14, 100%) yielded V3-V4 region amplicons, as did most (6/7, 85.71%) normal livers at 15 months. The pool of five worms also yielded PCR amplicons for 16S rRNA gene. In contrast, after culture in Brucella broth, all liver samples and pooled worms gave negative results for 16S RNA gene.

### Number of OTUs in different samples

Samples for which amplification of the V3-V4 region was successful were pooled for each experimental group and used for metagenomic analysis. Of 855,046 sequences, 417,953 with useable reads were assignable to 93 operational taxonomic units (OTUs) consisting of 80 OTUs of bacteria, 12 OTUs of hamster DNA, and one OTU of plant origin. In addition, one OTU was identified as a chimera (DNA of hamster + *Helicobacter* spp.). The presence of twelve OTUs of hamster and one chimera may be because the bacterial 16S gene is homologous with the mitochondrial 16S gene of animals and has some regions of sequence similarity. The 80 bacterial OTUs were assigned to 6 phyla and 42 genera (S3 Fig). Sequences of *Bifidobacterium* (GenBank accession number KX833862), *Escherichia* (KX833831) and *Helicobacter* (KX833878) were found only in the OV-infected group. OTUs of *Fusobacterium* (KX833856), *Aggregatibacter* (KX833819), *Streptococcus* (KX833876) and *Veillonella* (KX833867) were more abundant in the OV-infected group than in normal controls. In contrast, OTUs of *Acidaminococcus* (KX833858), *Clostridium* (KX833857), *Lactobacillus* (KX833859) and *Megasphaera* (KX833874) were less abundant in the OV-infected group compared to normal controls. Although fewer sequences were obtained from the OV-infected group, this group contained a greater range of OTUs (Table 2). In addition, more than 20 genera of bacteria were placed in the “Others” category, represented by fewer than 0.5% of the total number of sequences. The different bacterial communities in the chronic OV-infected group and in the normal control group in shown in S1 Table.

**Table 2:** Number of read sequences in bacteria at the genus-level in hamster liver and worm samples obtained by next generation sequencing (Illumina MiSeq platform) of V3-V4 hypervariable regions of prokaryotic 16S rDNA.

Genus-level	Number of read sequences or tags			
	Normal, liver	OV-infected, liver		Adult *O*. *viverrini*
	Thioglycollate	Frozen, PCR-positive C97-98	Thioglycollate	Thiogly-collate
*Acidaminococcus*	20221	1	3	0
*Aggregatibacter*	2233	15	3205	22853
*Bifidobacterium*	0	3	560	0
*Clostridium*	21742	0	6	2
*Escherichia*	0	3	9820	0
*Fusobacterium*	5	3	13314	0
*Helicobacter*	0	962	0	0
*Lactobacillus*	206694	39	23905	34744
*Megasphaera*	1704	0	1	1
*Streptococcus*	5279	11	10378	1
*Veillonella*	374	3	1244	0
Unclassified	4848	28724	3333	23
Other (<0.5%)	810	262	624	0
Total reads	263910	30026	66393	57624
Number of OTUs	40	52	37	9

Sequences of *Helicobacter* species were only obtained from some frozen, infected livers. Sequence comparisons in GenBank revealed 100% identity with *H*. *pylori*. Substantial levels of similarity were seen with other *Helicobacter* spp. However, it is unclear whether any of these species was actually represented in our materials.

### Alteration of microbiota in the liver after chronic *O*. *viverrini* infection

**Fig 2:**
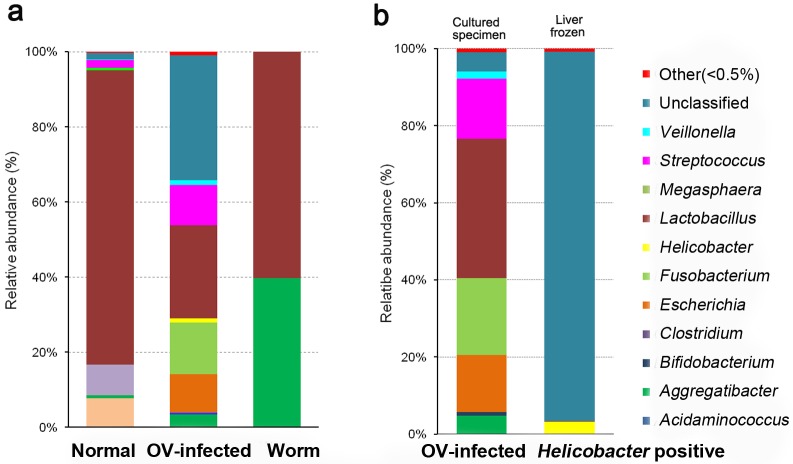
Distribution and diversity of bacterial DNA at genus-level in hamster liver and worm samples. (a) Normal, OV-infected and worm groups. (b) OV-infected group (cultured specimen vs. frozen liver). Genera accounting for less than 0.5% of sequences in all samples were classified into 'others'. Normal: Normal group, OV: *O*. *viverrini*-infected group, Worm: *O*. *viverrini* adult worms.

The phyletic distribution and relative abundances of bacterial sequences obtained at the genus level from hamster liver and hepatobiliary tract (HB) is shown in Fig 2. In the normal group, *Lactobacillus* (78.32%), *Clostridium* (8.24%), *Acidaminococcus* (7.66%), *Streptococcus* (2%), *Aggregatibacter* (0.85%), *Megasphaera* (0.64%), *Veillonella* (0.14%), unclassified (1.83%), and others (0.3%) were represented. In the chronic OV-infected group, the bacterial sequences present were from *Lactobacillus* (24.83%), *Fusobacterium* (13.81%), *Streptococcus* (10.77%), *Escherichia* (10.19%), *Aggregatibacter* (3.34%), *Veillonella* (1.29%), *Helicobacter* (0.99%), *Bifidobacterium* (0.58%), *Clostridium* (0.006%), unclassified (33.24%), and others (0.92%). In adult worms, only *Aggregatibacter* (39.65%), and *Lactobacillus* (60.29%) were identified as shown in Fig 2a. The phyletic distribution and relative abundances of bacterial sequences in cultured samples from chronic OV-infected hamsters were *Lactobacillus* (36.01%), *Fusobacterium* (20.05%), *Streptococcus* (15.63%), *Escherichia* (14.79%), *Aggregatibacter* (4.83%), *Veillonella* (1.87%), *Bifidobacterium* (0.84%), *Clostridium* (0.009%), unclassified (5.02%), and others (0.94%) (Fig 2b). Among the *Helicobacter*-positive frozen samples from the chronic OV-infected group, the great majority of sequences could not be classified (95.66%). Identifiable taxa included *Helicobacter* (3.20%), *Lactobacillus* (0.13%), unclassified (95.66%), and others (0.88%, Fig 2b). Notably, there was a greater bacterial diversity at the genus-level in OV-infected hamsters than in the normal group or in adult worms (Table 2).

**Fig 3:**
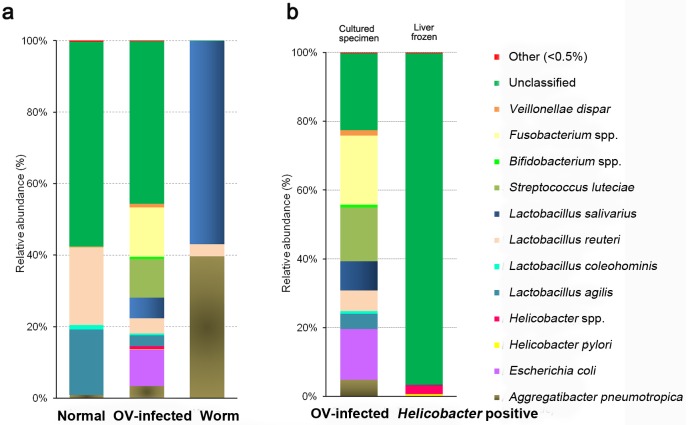
Distribution and diversity of bacterial DNA at species-level in hamster livers and worm samples. (a) Normal, OV-infected and worm groups. (b) OV-infected group (cultured specimen vs. frozen liver). Species accounting for less than 0.5% of sequences in all samples were classified into 'others'. Normal: Normal group, OV: *O*. *viverrini*-infected group, Worm: *O*. *viverrini* adult.

The diversity of bacterial taxa at the species level is shown in Fig 3. *Lactobacillus reuteri* (GenBank accession number KX833821) (21.55%), *L*. *agilis* (KX833820) (18.23%), *L*. *coleohominis* (KX833829) (1.26%), *Aggregatibacter pneumotropica* (KX833819) (0.84%), *Veillonella dispar* (KX833830) (0.1%), *Streptococcus luteciae* (KX833832) (0.08%) and *Lactobacillus salivarius* (KX833822) (0.02%), were identified in normal liver tissues. In the chronic OV-infected group, *Fusobacterium* spp. (13.81%), *S*. *luteciae* (10.76%), *Escherichia coli* (KX833831) (10.18%), *L*. *salivarius* (5.85%), *L*. *reuteri* (4.16%), *A*. *pneumotropica* (3.33%), *L*. *agilis* (3.02%), *V*. *dispar* (1.09%), *Helicobacter* spp. (0.82%), *Bifidobacterium* spp. (0.58%), *L*. *coleohominis* (0.56%), *Helicobacter pylori* (KX833824) (0.17%) and unclassified (45.36%) were present. In adult worms, three species were identified, *L*. *salivarius* (56.85%), *A*. *pneumotropica* (39.66%), and *L*. *reuteri* (3.44%) (Fig 3a). In the frozen material from the OV-infected group, when considering only *Helicobacter*-positive samples from frozen liver, again unclassified sequences made up the great majority (96.34%). Other species represented were *Helicobacter* spp. (2.64%), *H*. *pylori* (0.56%), unclassified (96.34%), and others (0.22%) (Fig 3b). Cultured (thioglycollate broth) bacterial communities from normal livers, infected livers and worms varied substantially. The highest number of bacterial species was seen in the chronic OV-infected group. Notably, in adult worms, sequences of *L*. *salivarius* were abundant (56.84%), but *H*. *pylori* wasn’t detected.

### Changes of bacterial community after chronic *O*. *viverrini* infection

**Fig 4:**
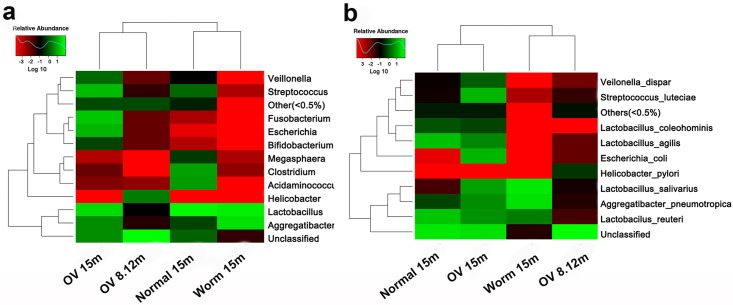
Heat map of identified bacteria at genus (a) and certain species (b) levels in hamster livers and adult worms. Rows indicate the abundance of sequences from each taxon among different samples, and columns indicate the abundance of sequences from each taxon within each sample. The tree at the left-hand side of each heat map clusters rows according their similarity and the tree above each heat map groups samples by their similarity. Normal. 15 m: Normal group (n = 7, pooled), OV.15m: *O*. *viverrini*-infected group at 15 months (n = 8, pooled), OV.8.12m: *O*. *viverrini*-infected groups at 8 and 12 months (n = 6, pooled), Worm 15 m: worms obtained from *O*. *viverrini*-infected hamsters 15 months p.i. (n = 2 pooled)

Fig 4 shows the taxonomic clustering of microbiomes based on 16S rDNA sequences from liver and worms according to time post-infection. *Aggregatibacter*, *Lactobacillus* and unclassified were similar in abundance among the three liver groups. Sequences of *Fusobacterium*, *Escherichia* and *Bifidobacterium* were at highest abundance in the OV-infected groups. In contrast, *Megasphaera*, *Clostridium*, and *Acidaminococcus* were most abundant in normal livers, but were less common in the OV-infected groups and in adult worms. *Lactobacillus salivarius* was most abundant in worms (56.84%) and in OV-infected hamsters at 15 months (8.461%).

**Fig 5:**
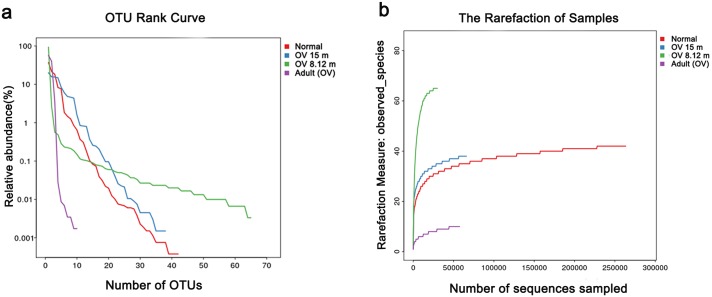
Complexity of microbial diversity in hamster liver and worm samples in chronic opisthorchiasis. (a) OTU rank abundance curve. (b) Rarefaction plot of alpha diversity (observed species indices).

The community diversity in microbiota of OV-infected liver was analyzed by OTU rank curve, observed species and Shannon indices (Fig 5). The OTU rank abundance curve (Fig 5a) provides a means for visually representing species richness and evenness. The steepest slope was seen in the 15-month adult *O*. *viverrini* group showing that a small number of genera accounted for most of the abundance. In the OV-infected 8, 12 month group (frozen livers), the shallower slope implies a greater degree of species evenness. The rarefaction curves (Fig 5b) were approaching an asymptote in all groups, suggesting that few additional taxa remained to be sampled.

**Fig 6:**
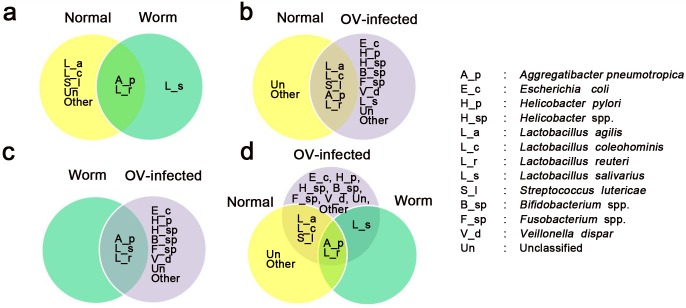
Venn diagram of identified bacterial species among different groups. (a) Normal group and adult worm group. (b) Normal group and OV-infected group. (c) Worm group and OV-infected group. (d) Among three groups.

A Venn diagram was constructed (Fig 6) to show the extent of sharing of bacterial taxa by the different groups. Species shared by normal liver and worms were *A*. *pneumotropica* and *L*. *reuteri*, while *L*. *salivarius* was identified in worms only. *Lactobacillus agilis*, *L*. *coleohominis*, *S*. *luteciae*, *A*. *pneumotropica* and *L*. *reuteri* were isolated from normal and OV-infected groups, suggesting that these bacteria are normal flora in the hepatobiliary system. The greatest bacterial diversity was found in the infected groups. *V*. *dispar* and *L*. *salivarius* were found only in the infected group, suggesting that *O*. *viverrini* infection increases gut microbiota and might enhance bacterial recruitment to other sites. *Aggregatibacter pneumotropica*, *L*. *salivarius* and *L*. *reuteri* were identified from worms and from the OV-infected group, implying that worms might become infected while residing in the hepatobiliary system of the host. Notably, *A*. *pneumotropica* and *L*. *reuteri* were found in all three groups, suggesting that these two species are normal flora in the hepatobiliary tracts of hamsters. Moreover, *E*. *coli*, *H*. *pylori*, *Helicobacter* spp., *Bifidobacterium* spp., *Fusobacterium* spp., and *V*. *dispar* were identified only in the OV-infected group.

### Identification of *Helicobacter pylori* by species-specific PCR (*ureA* gene) from liver tissue

We identified the presence of *H*. *pylori* by PCR using species-specific primers. Only one of the three *Helicobacter* genus-positive samples from the OV-infected group yielded amplicons for the *ureA* gene, which is consistent with the metagenomics result. The liver sample positive by PCR was the same sample yielding the positive immunostaining result. The amplified product of the *ureA* gene from this sample is shown in S4 Fig. In addition, in ongoing work, we have found worms themselves to be PCR-positive for *H*. *pylori*, similar to the immunohistochemistry result.

### The presence of *Helicobacter pylori* infection in tissue and worms demonstrated by Immunostaining

**Fig 7:**
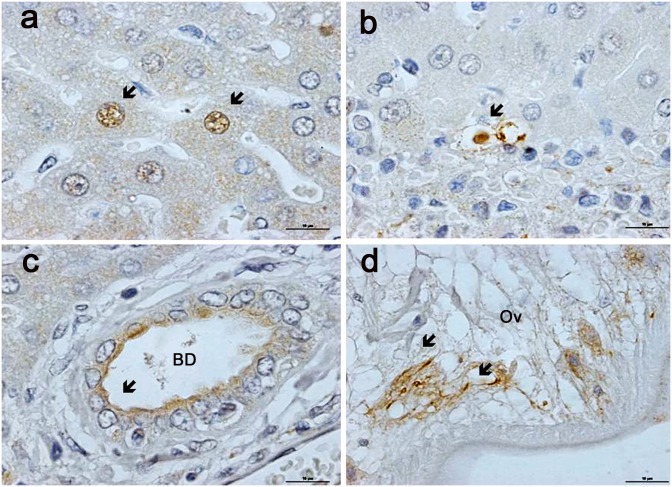
Immunohistochemical localization of *Helicobacter pylori* infection in hamster liver infected with *O*. *viverrini*. Immunostaining for *H*. *pylori* appears as brown color (arrow) within (a) hepatocytes, (b) sinusoid, (c) bile duct and (d) *O*. *viverrini* adult worm. Original magnification, x1000, Scale bar is 10μm.

To verify the PCR result, we applied immunohistochemistry to the three samples that were positive for genus-specific primers (C97&C98) by PCR, and to three negative samples for comparison. Only the single sample from which the *ureA* gene had been amplified proved positive for *H*. *pylori* by immunostaining. Immunoreactivity revealed that *H*. *pylori* was present in hepatocytes, sinusoids and epithelial cells of a large bile duct, and inside the *O*. *viverrini* worm. No immunoreactivity was seen in normal liver (Fig 7).

## Discussion

Bacterial infection has previously been implicated in hepatobiliary diseases (HBDs) in human [12, 31] and hamster tissues [32]. Boonyanugomol et al. have identified *H*. *pylori* in CCA tissues [12]. In order to search for bacteria associated with HBD caused by chronic opisthorchiasis in hamster livers, here we investigated a microbial community using metagenomic analysis. Bile was not available from hamsters chronically infected with *O*. *vivverrini* due to worm obstruction of the bile ducts and decreasing bile synthesis [33]. However, the bile duct tissue itself could still contain a microbial community in the liver. Moreover, although amplification of V3-V4 hypervariable regions of prokaryotic 16S rRNA gene from fresh liver tissues was not successful, we were able to identify the bacterial microbiome in material cultured from the liver. This may be due to bacteria mainly residing in residual bile rather than in liver tissue. The presence of *Helicobacter pylori* mainly in bile and sinusoids, rather than in the liver cells (Fig 7), may support this explanation. However, *H*. *pylori* was successfully demonstrated in the liver tissue and worms by immunostaining, suggesting chance occurrence in some samples.

In this study, based on cultured material and high-throughput next-generation sequencing (NGS) coupled with the metagenomics study of 16S rDNA sequences (V3-V4 regions), 417,953 reads were assignable to 93 operational taxonomic units (OTUs) consisting of 80 OTUs of bacteria (6 phyla and 42 genera) in the liver and in adult worms. Contamination by hamster and plant DNA is not unexpected and must explain the finding of hamster and plant sequences. Enrichment of microbes in *O*. *viverrini*-infected livers (at 8, 12, and 15 months p.i.) revealed that the most common phyla of bacteria represented were *Proteobacteria* (16 genera), *Firmicutes* (15 genera), *Actinobacteria* (6 genera), *Bacteroidetes* (3 genera), and a single genus for each of *Cyanobacteria* and *Fusobacterium*. Sequences of *Fusobacterium* spp. had the highest relative abundance (13.81%), followed by *Streptococcus luteciae* (10.76%), *Escherichia coli* (10.18%) and *Bifidobacterium* spp. (0.58%). Much higher microbial diversity was detected in cultured samples from infected livers than in similar samples from normal livers. The rank abundance and rarefaction curves suggest there are more species of bacteria in the *O*. *viverrini*-infected group than in normal controls (Fig 5). Thus, *H*. *pylori* (0.17%) and *Helicobacter* spp. (0.82%) were identified in the livers of hamsters with chronic opisthorchiasis, but not in normal livers. However, it should be noted that use of real-time PCR might detect *Helicobacter* in normal livers as previously reported [34].

Several species of *Helicobacter* have been identified in normal hamsters at 18 to 24 months of age [32, 35–37]. Although *Helicobacter* spp. are usually resident in the gastrointestinal tract (GI) of rodents [32], they can also be harbored by worms in the host [34], resulting in their presence in the bile duct lumen and worms (Fig 7). In cultured material, DNA from *Aggregatibacter pneumotropica*, *Lactobacillus reuteri* and *L*. *salivarius* was commonly found in the chronic OV-infected group. These results suggest that worms may carry bacteria from the GI system into the hepatobiliary system in chronic opisthorchiasis. In support of this, two species (*A*. *pneumotropica*, *L*. *reuteri*) of bacteria could be identified in normal livers as well as in worms (Fig 6) and a third, *L*. *salivarius*, was found in the OV-infected group as well as in worms.

In agreement with earlier studies, we identified several bacteria species, including aerobic, microaerophilic and anaerobic bacteria, in HBD after culture in thioglycollate. The most commonly found aerobic bacteria were *Escherichia coli*, and *Enterobacter* spp. In addition, anaerobic bacteria including *Bacteroides*, *Clostridium* and *Fusobacterium* were also frequently identified. Several microorganisms have been associated with carcinogenesis. For instance, *H*. *pylori*, *E*. *coli*, *Fusobacterium*, and Enterobacteriaceae have been implicated in colorectal carcinoma [38, 39]. Among microaerophilic bacteria, members of the genus *Helicobacter* are the most interesting, because they have been implicated in a variety of GI diseases including peptic ulcer, gastric cancer, and inflammatory bowel disease in humans and animals [32, 35, 37, 40]. Moreover, *H*. *pylori* is well known as a causative agent in gastric cancer [41]. We postulate that *H*. *pylori* and closely related *Helicobacter* spp. might contribute to induction of hepatobiliary disease during chronic *O*. *viverrini* infection.

More than 20 genera of bacteria were placed in the “Others” category in the chronic OV-infected group. Each genus was represented by fewer than 0.5% of the total number of sequences (S1 Table). Probiotic bacteria such as *Lactobacillus* are useful in healthy humans, reducing the risk of developing diseases [42]. Interestingly, our study demonstrated that probiotic bacteria were in higher abundance in normal livers than in the chronic OV-infected group. In the latter group, greater numbers of pathogenic bacteria such as *E*. *coli*, *H*. *pylori*, *Helicobacter* spp., *Streptococcus luteciae*, *Bifidobacterium* spp., and *Fusobacterium* spp., were seen in the liver and fewer *L*. *reuteri*, *L*. *agilis*, *L*. *coleohominis*. We suggest that a predominance of some gastrointestinal pathogenic bacteria and a decrease in beneficial bacterial species during *O*. *viverrini* infection might induce HBD. The changes in liver microbiome are analogous to those seen in Crohn's disease patients [43]. Unexpectedly, *L*. *salivarius* was found abundantly in adult worms (56.84%) and in OV-infected hamsters at 15 months p.i. (8.5%). In these cases, however, *H*. *pylori* was not detected, suggesting *L*. *salivarius* suppresses *H*. *pylori* viability and reduces the risk of *H*. *pylori* infection [44].

*Helicobacter pylori*, a carcinogenic agent has been identified in gastric cancer and also in CCA tissues [12]. We therefore thought it important to confirm metagenomic results using PCR and immunohistochemistry techniques. The presence of *H*. *pylori* and non-*Helicobacter* species in the liver during chronic opisthorchiasis might reflect obstruction of the biliary system due to the presence of adult worms, and a consequent influx of bacterial growth from the GI tract. A chemotactic response of *H*. *pylori* to bile [45] due to the reflux of bile into the stomach leads to enhanced growth and its colonization into the hepatobiliary system during chronic infection. Reflux of bile into the stomach postprandially is common in patients, especially those with gastroduodenal disease. Thus, a high prevalence of *H*. *pylori* was found in ulcers of cirrhotic patients [46]. Alternatively, *O*. *viverrini* may serve as a reservoir or vector for *Helicobacter* [34], leading to development of HBD during liver fluke infection. We could not identify *H*. *pylori* in adult worms using metagenomic analysis, but could detect it using in-situ hybridization, suggesting that the presence of *H*. *pylori* in worms, constituting fewer than 1% of sequences, is only an occasional occurrence.

A similar suite of bacteria, such as *Helicobacter*, Enterobacteriaceae, *Bacteroides*, *Lactobacillus* and anaerobic bacteria in the livers of hamsters with chronic opisthorchiasis, was found using molecular methods in CCA tissues of patients [47]. Also, in non-liver fluke endemic areas, bacteria cause most cases of infectious cholangitis in Western countries [48]. Thus, these bacteria may enter the biliary tree and cause cholangitis leading to HBD which finally might develop into CCA. We must concede that the liver microbiome data reported in this study may have been subject to bias because it depended on culture-enriched bacteria: bacterial species that do not grow in thioglycollate broth would not be represented. Moreover, *H*. *pylori* was only verified in one sample using immunohistochemistry (n = 1) and further work is required to confirm the significance of this finding. Also, a final conclusion requires further investigation of the bacterial community in bile duct disease-associated liver fluke infection.

**In conclusion,** based on a metagenomics study of 16S rDNA sequences (V3-V4 regions) obtained by next-generation sequencing of bacteria cultured from livers and from adult worms, we have successfully identified the microbiota in liver tissue after culture-enrichment of bacteria. *Helicobacter pylori* and non-*Helicobacter* spp. were also identified in the liver in chronic opisthorchiasis. We suggest that chronic opisthorchiasis enhances bacterial diversity in the liver, which might be related to development of HBD.

## Supporting Information

S1 Fig Representative gel image of PCR results using primers for the V3-V4 region of prokaryotic 16S rDNA. Liver frozen; Lane 3: OV-infected 1 month, Lane 4: Normal 4 months, Lane 5: OV-infected 4 months, Lane 6: Normal 8 months, Lane 7: OV-infected 8 months, Lane 8: OV-infected 12 months. Cultured materials; Lane 9–12: OV-infected 8 months, Lane 13–14: OV-infected 12 months, M = marker, P = positive control *H*. *pylori*, N = negative control.
(DOCX)

S2 Fig Representative gel image of PCR results using primers for the V3-V4 region of prokaryotic 16S rDNA. The template DNA was PCR reaction products from frozen samples that were positive with *Helicobacter* genus-specific primers. Lane 1–8: OV-infected 8 & 12 months, M = marker, P = positive control *H*. *pylori*, N = negative control.
(DOCX)

S3 Fig Phylogenetic tree of identified bacteria genera associated with chronic opisthorchiasis based on the nucleotide sequences of the V3-V4 hypervariable region of prokaryotic 16S rDNA. Phyla are color-coded according to the key on the right.
(DOCX)

S4 Fig Detection of *Helicobacter pylori* (*ureA* gene) from frozen liver specimens by PCR. Product size was 350 bp. M: 100-bp molecular weight marker, P: Positive control, N: Negative control, Lanes 1–3: samples that were positive with genus-specific primers for *Helicobacter* were subsequently subjected to PCR using the *H*. *pylori*- specific primers for the *ureA* gene. Only in lane 1 did the *H*. *pylori*-specific primers produce a positive result.
(DOCX)

S1 Table Numbers of read sequences from bacteria (identified to genus-level) cultured from normal livers, OV-infected livers and adult worms. Only genera represented by <0.5% of sequences in all samples are included. Sequences were obtained by next generation sequencing (Illumina MiSeq platform) of the V3-V4 hypervariable regions of prokaryotic 16S rDNA.
(DOCX)

## Abbreviations

OV: *Opisthorchis viverrini*
CCA: cholangiocarcinoma
HBD: hepatobiliary disease
PCR: polymerase chain reaction
rDNA: ribosomal DNA
OTU: Operational Taxonomic Unit
RDP: Ribosomal Database Project
