# Supplementary material for: Chronic Opisthorchis viverrini Infection Changes the Liver Microbiome and Promotes Helicobacter Growth
Source: PLoS One. 2016 Nov 2;11(11):e0165798. doi: 10.1371/journal.pone.0165798 (PMC5091914; doi:10.1371/journal.pone.0165798)
Supplement: S1 Fig — Liver frozen; Lane 3: OV-infected 1 month, Lane 4: Normal 4 months, Lane 5: OV-infected 4 months, Lane 6: Normal 8 months, Lane 7: OV-infected 8 months, Lane 8: OV-infected 12 months. Cultured materials; Lane 9–12: OV-infected 8 months, Lane 13–14: OV-infected 12 months, M = marker, P = positive control H. pylori, N = negative control. (DOCX) [file pone.0165798.s001.docx]

**Supporting Information**

**S1 Fig.** **Representative gel image of PCR results using primers for the V3-V4 region of prokaryotic 16S rDNA.**  Liver frozen; Lane 3: OV-infected 1 month, Lane 4: Normal 4 months, Lane 5: OV-infected 4 months, Lane 6: Normal 8 months, Lane 7: OV-infected 8 months, Lane 8: OV-infected 12 months. Cultured materials; Lane 9-12: OV-infected 8 months, Lane 13-14: OV-infected 12 months, M = marker, P = positive control *H. pylori*, N = negative control.
