# Supplementary material for: Chronic Opisthorchis viverrini Infection Changes the Liver Microbiome and Promotes Helicobacter Growth
Source: PLoS One. 2016 Nov 2;11(11):e0165798. doi: 10.1371/journal.pone.0165798 (PMC5091914; doi:10.1371/journal.pone.0165798)
Supplement: S2 Fig — The template DNA was PCR reaction products from frozen samples that were positive with Helicobacter genus-specific primers. Lane 1–8: OV-infected 8 & 12 months, M = marker, P = positive control H. pylori, N = negative control. (DOCX) [file pone.0165798.s002.docx]

**Supporting Information**

**S2 Fig. Representative gel image of PCR results using primers for the V3-V4 region of prokaryotic 16S rDNA.** The template DNA was PCR reaction products from frozen samples that were positive with *Helicobacter* genus-specific primers. Lane 1-8: OV-infected 8 & 12 months, M = marker, P = positive control *H. pylori*, N = negative control.
