# Supplementary material for: Chronic Opisthorchis viverrini Infection Changes the Liver Microbiome and Promotes Helicobacter Growth
Source: PLoS One. 2016 Nov 2;11(11):e0165798. doi: 10.1371/journal.pone.0165798 (PMC5091914; doi:10.1371/journal.pone.0165798)
Supplement: S3 Fig — Phyla are color-coded according to the key on the right. (DOCX) [file pone.0165798.s003.docx]

**Supporting Information**

**S3 Fig. Phylogenetic tree of identified bacteria genera associated with chronic opisthorchiasis based on the nucleotide sequences of the V3-V4 hypervariable region of prokaryotic 16S rDNA.** Phyla are color-coded according to the key on the right.
