# Supplementary material for: Chronic Opisthorchis viverrini Infection Changes the Liver Microbiome and Promotes Helicobacter Growth
Source: PLoS One. 2016 Nov 2;11(11):e0165798. doi: 10.1371/journal.pone.0165798 (PMC5091914; doi:10.1371/journal.pone.0165798)
Supplement: S4 Fig — Product size was 350 bp. M: 100-bp molecular weight marker, P: Positive control, N: Negative control, Lanes 1–3: samples that were positive with genus-specific primers for Helicobacter were subsequently subjected to PCR using the H. pylori- specific primers for the ureA gene. Only in lane 1 did the H. pylori-specific primers produce a positive result. (DOCX) [file pone.0165798.s004.docx]

**Supporting Information**

**S4 Fig.**  **Detection of *Helicobacter pylori* (*ureA* gene) from frozen liver specimens by PCR.** Product size was 350 bp. M: 100-bp molecular weight marker, P: Positive control, N: Negative control, Lanes 1-3: samples that were positive with genus-specific primers for *Helicobacter* were subsequently subjected to PCR using the *H. pylori*- specific primers for the *ure*A gene. Only in lane 1 did the *H. pylori*-specific primers produce a positive result.
