# Supplementary material for: Chronic Opisthorchis viverrini Infection Changes the Liver Microbiome and Promotes Helicobacter Growth
Source: PLoS One. 2016 Nov 2;11(11):e0165798. doi: 10.1371/journal.pone.0165798 (PMC5091914; doi:10.1371/journal.pone.0165798)
Supplement: S1 Table — Only genera represented by <0.5% of sequences in all samples are included. Sequences were obtained by next generation sequencing (Illumina MiSeq platform) of the V3-V4 hypervariable regions of prokaryotic 16S rDNA. (DOCX) [file pone.0165798.s005.docx]

**Supporting Information**

**S1 Table. Numbers of read sequences from bacteria (identified to genus-level) cultured from normal livers, OV-infected livers and adult worms.** Only genera represented by <0.5% of sequences in all samples are included. Sequences were obtained by next generation sequencing (Illumina MiSeq platform) of the V3-V4 hypervariable regions of prokaryotic 16S rDNA.

|  |  | **Number of useable reads** | | |
| --- | --- | --- | --- | --- |
| **Genus** | **GenBank Accession number** | **Normal** | **OV-infected** | **Adult**  ***O. viverrini*** |
| *Varibaculum* | KX833853 | 15 | 0 | 0 |
| *Corynebacterium* * | KX833841 | 0 | 3 | 0 |
| *Kocuria* * | KX833818 | 0 | 7 | 0 |
| *Pimelobacter* * | KX833870 | 0 | 2 | 0 |
| *Atopobium* * | KX833860 | 18 | 136 | 0 |
| *Bacteroides* * | KX833833 | 0 | 3 | 0 |
| *Parabacteroides* * | KX833827 | 52 | 93 | 0 |
| *Prevotella* | KX833854 | 16 | 1 | 0 |
| *Chroococcidiopsis* * | KX833848 | 0 | 18 | 0 |
| *Staphylococcus* | KX833847 | 46 | 3 | 0 |
| *Granulicatella* * | KX833846 | 4 | 64 | 0 |
| *Oribacterium* * | KX833864 | 104 | 312 | 0 |
| *Ruminococcus* * | KX833826 | 0 | 64 | 0 |
| *Peptostreptococcus* | KX833825 | 478 | 14 | 0 |
| *Finegoldia* * | KX833840 | 0 | 5 | 0 |
| *Peptoniphilus* | KX833850 | 33 | 0 | 0 |
| *Dialister* | KX833880 | 2 | 1 | 0 |
| *Allobaculum* | KX833837 | 10 | 0 | 0 |
| *Brevundimonas* * | KX833816 | 0 | 7 | 0 |
| *Balneimonas* * | KX833838 | 0 | 5 | 0 |
| *Agrobacterium* * | KX833851 | 1 | 15 | 0 |
| *Skermanella* * | KX833873 | 0 | 4 | 0 |
| *Sutterella* | KX833879 | 6 | 5 | 0 |
| *Comamonas* * | KX833845 | 0 | 10 | 0 |
| *Hydrogenophilus* * | KX833865 | 2 | 31 | 0 |
| *Pasteurella* | KX833866 | 16 | 0 | 0 |
| *Acinetobacter* * | KX833844 | 0 | 34 | 0 |
| *Pseudomonas* * | KX833834 | 0 | 27 | 0 |
| *Pseudoxanthomonas* * | KX833828 | 0 | 2 | 0 |
| *Stenotrophomonas* * | KX833868 | 1 | 7 | 0 |

* Indicates genera for which numbers of sequences were much higher in OV-infected hamster livers than in normal livers.
